# Epidemiology of pharyngitis as reported by Zambian school children and their families: implications for demand-side interventions to prevent rheumatic heart disease

**DOI:** 10.1186/s12879-017-2563-x

**Published:** 2017-07-06

**Authors:** John Musuku, Joyce C. Lungu, Elizabeth Machila, Catherine Jones, Laurence Colin, Sherri Schwaninger, Patrick Musonda, Brigitta Tadmor, Jonathan M. Spector, Mark E. Engel, Liesl J. Zühlke

**Affiliations:** 1 University Teaching Hospital, Nationalist Rd, Lusaka, Zambia; 2 Novartis Institutes for BioMedical Research, 5300 Chiron Way, Emeryville, CA 94608 USA; 3 Novartis Institutes for BioMedical Research, 250 Massachusetts Ave, Cambridge, MA 02139 USA; 4 University of Zambia, Great E, Lusaka, Zambia; 5 Department of Medicine, University of Cape Town, Rondebosch, Cape Town, 7700 South Africa; 6 Department of Paediatrics, Red Cross War Memorials Children’s Hospital, University of Cape Town, Cape Town, South Africa

**Keywords:** Pharyngitis, Rheumatic heart disease, Zambia, Cross-sectional study

## Abstract

**Background:**

Prompt and appropriate treatment of streptococcal pharyngitis decreases the risk of acute rheumatic fever and rheumatic heart disease (RHD). Understanding public perceptions and behaviors related to sore throat is fundamental to inform health programs aimed at eliminating new cases of RHD in endemic regions. We sought to describe the epidemiology of pediatric pharyngitis and its treatment, as reported by children and their parents or guardians in Lusaka, Zambia.

**Methods:**

This was a cross-sectional investigation using interviews and written surveys, nested in a school-based RHD prevalence study. Students and their parents were asked to report number of sore throats in the previous 12 months, treatment received, and type and place of treatment. A focused history and physical examination to detect pharyngitis was conducted and children were referred for follow-up as indicated.

**Results:**

A total of 3462 students from 47 schools participated in the study, along with their parents or guardians. Six hundred and fifty eight (19%) parents/guardians reported their child had at least one sore throat in the previous year, and 835 (24%) of students reported at least one sore throat in the same time period. Girls were reported to have pharyngitis 50% more often than boys, and also made up two-thirds of the total students treated. Approximately two-thirds of children who had at least one episode of pharyngitis during the previous year were also reported to have received some form of treatment. The majority of treatments were received in government clinics (36.6%) and at home (26.3%). Half of treatments included an antibiotic. Nineteen students (0.5%) had clinically-apparent pharyngitis at screening.

**Conclusion:**

Pharyngitis is common among school-aged children and adolescents in Zambia, with females reporting significantly more sore throat episodes than males. Parents/guardians have variable knowledge about the frequency of sore throat in their children, and management of pharyngitis may be suboptimal for many children since more than a quarter were reported to have received treatment without skilled assessment. These results provide insight into current perceptions and practices related to sore throat in Zambia and will be used to design public awareness activities aimed at reducing RHD.

## Background

Rheumatic heart disease (RHD) is a potential complication of untreated group A β-haemolytic streptococcal (GAS) pharyngitis that is endemic in many low- and middle-income countries and in some indigenous populations of high-income countries [1, 2]. RHD is a disease of poverty and, as a reflection of its enduring neglect, robust assessments of its prevalence were reported for the first time only several years ago. The Global Burden of Disease Study (2015) estimated that more than 33 million people are living with RHD [3] and 320,000 people die of the disease each year [4]. In sub-Saharan Africa, population-based screening studies using portable echocardiography have consistently revealed rates of 1–3% in schoolchildren [5–8].

Rheumatic heart disease is the consequence of an aberrant immune response following GAS pharyngitis, whereby antibodies elicited to the bacterial pathogen cross react with proteins in the heart [1]. If not treated appropriately, GAS pharyngitis leads in a small percentage of patients to acute rheumatic fever (ARF). Rheumatic heart disease develops in nearly half of patients with ARF and is typically characterized by progressive damage to heart valves spurred by repeat exposures to GAS. Patients with RHD are at risk of debilitating heart failure, stroke, endocarditis, and premature death [1].

New appreciation of the substantial burden of RHD has led to expansion of health policy, programming, and research aimed at combating the disease in endemic regions [9–11]. A major focus is disease prevention. In this regard, prompt and correct treatment of streptococcal pharyngitis with antibiotics, known as primary prevention, has emerged as a critical priority since it is the singular clinical activity known to decrease attack rates of ARF and RHD [12, 13]. Primary prevention was recognized to be a key component of successful national public health initiatives in several countries [14, 15], and it is highlighted as an essential practice by leading health authorities including the World Health Organization, the World Heart Federation, and the Pan African Society for Cardiology [16, 17]. As a result, concerted efforts are underway in various parts of the world, including in sub-Saharan Africa, to scale-up health workers’ capability to satisfactorily manage patients that present with sore throat [9, 10].

GAS pharyngitis should be treated with antibiotics within 9 days from onset to eliminate the risk of RHD [18], and it is therefore incumbent on patients to promptly seek skilled care when symptoms manifest. Yet while the public’s role in anti-RHD efforts is paramount, few if any large-scale RHD initiatives address the demand-side barriers related to health seeking behaviors and patterns of health care utilization that are common in settings where the disease is prevalent. “Demand-side barriers” are defined here as determinants of health care utilization that operate at the individual, household, or community level; they include educational, cultural, and social factors [19]. As part of a multi-faceted public-private partnership in Zambia to control and eventually eliminate RHD, an awareness campaign was launched in 2014 with the primary aim of educating the public about the link between pharyngitis and RHD. In order to inform relevant and effective campaign messaging, we sought to understand baseline perceptions and behaviors related to sore throat. A large-scale school-based screening program designed to measure RHD prevalence provided the opportunity to nest an investigation of the epidemiology of pediatric pharyngitis and its treatment as reported by children and their parents or guardians.

## Methods

### Study population

The study population consisted of healthy children and young adults in Zambia’s capital, Lusaka, between September 2014 and November 2015. Based upon methods used previously﻿ [8], the population was drawn from a random sample of grade 1–12 classrooms in public primary and secondary schools. A study team member approached the administrative head of each selected school, discussed the proposed study, and invited participation. Study staff then also briefed teachers whose classrooms were to be involved. The parents or guardians of study participants provided written informed consent. In addition, written informed assent was required from participants 8 years of age or older. Inclusion criteria were all students who provided assent, whose parents consented, and who were present at school on the day of the study. Exclusion criteria were conditions that precluded the performance of transthoracic echocardiography that was required for the disease prevalence aspect of the study (e.g., skin infection of the chest). This investigation was conducted in partnership with the Zambian Ministries of Health and Education. Ethics approval was granted from the University of Zambia.

### Data collection and management

This was an observational, cross-sectional investigation using interviews and written surveys that was nested in an RHD prevalence study. As part of the prevalence study component (analyses in progress) a clinical nurse practitioner examined students to detect signs of ARF or RHD, and a trained technician performed a screening echocardiogram to identify individuals with possible clinical or subclinical RHD. For the current investigation, students and their parents or guardians were interviewed or surveyed, respectively, and asked to report the number of episodes of sore throat that students had in the previous 12 months and whether or not treatment was received. If treatment was received, then efforts were made to understand the type of therapy (e.g., whether or not an antibiotic was administered) and the site of treatment. The Zambia national standard treatment guidelines recommend penicillin in either oral or intramuscular formulations to treat sore throat, and this is the most widely observed practice. This study did not attempt to describe what antibiotics specifically were administered for treatment of pharyngitis.

A clinical nurse practitioner conducted a focused history and physical examination of each student to detect for signs of pharyngitis at the time of the study. All students identified by the nurse practitioner to possibly have an abnormal clinical condition (including pharyngitis, ARF, or RHD) were referred for follow up at a local health clinic or at Lusaka University Teaching Hospital.

Data elements obtained by survey of parents or guardians were recorded initially on paper-based case report forms (by the parents or guardians themselves) and then transcribed by two study nurses into a secure, digital database (CommCare platform [20] an open-source, cloud-based software platform for health programs that supports the development of customized data-capture tools [21]). Data elements acquired by survey of students were obtained verbally and entered at the time of capture into the digital database using mobile electronic devices (Samsung Galaxy Tab. 2 computer tablets). Data relating to approximately 5% of subjects were verified between paper and electronic forms to validate accuracy of transcription. A data-extraction job was created to export data from CommCare into Microsoft Excel (Version 14.6.8) for analysis.

### Statistical analysis

The study was powered for the RHD prevalence aspect of the investigation. The sample size calculation took into account the primary and secondary school population in Lusaka (approximately 500,000), the point estimate of 1% RHD prevalence in Lusaka (based on previously conducted RHD prevalence studies from other countries in sub-Saharan Africa), a 1.2 design effect for clustering in school classrooms, and a presumed rejection rate of 9%. A sample size of at least 1024 participants was determined to provide an estimate of RHD prevalence within 0.7 percentage points of precision with 95% confidence. Ultimately, the number of students enrolled was greater than the minimum sample size because echocardiography-related methodological issues emerged after the study started and necessitated expansion of the number of subjects. That development had no effect on the current study other than enlarging the population studied, still under a randomization scheme.

Data analysis used the following methods: agreement between numbers of sore throats reported by parents and students was calculated using Cohen’s kappa, a commonly used statistic to determine inter-rater agreement for categorical variables. Linear regression was used to identify variables that could be predictors of the number of reported sore throats by children and parents, and stepwise regression was used to determine which variables to include in the model; statistically significant variables at the 15% level were added to the model one by one, and at each step previously added variables that were no longer found to be significant at the 15% level were removed once the new variable was added.

## Results

### Demographic characteristics

A total of 3462 children and adolescents participated, along with their parents or guardians. The families were enrolled at 47 different Lusaka area schools; approximately 60–90 students participated from each included school. The students ranged in age from 5 to 29 years, with a median of 14.0 years old (Table 1). The majority (98%) of students were ≤19 years of age. There were fifteen students ≥20 years of age (older students occasionally attend secondary school in Lusaka if their previous education had been disrupted by factors such as illness, pregnancy, childbirth, and need to work).

**Table 1:** Demographic characteristics of study participants (students)

Age (years)	Mean (SD)	14.09 (3.11)
	Median	14.0
Gender - (n%)	Male	1525 (44.04%)
	Female	1937 (55.93%)
Weight (kg)	Mean (SD)	45.8 (13.07)
	Median	47.4
Height (cm)	Mean (SD)	152.3 (14.37)
	Median	155.1

### Number of pharyngitis episodes as reported by student and parent or guardian

Twenty-four percent of students reported experiencing at least one sore throat in the previous year, with 4.6% reporting at least three episodes. According to student reports, the average number of sore throats in the previous year was 0.32 episodes per student. Nineteen percent of parents and guardians reported their child had at least one sore throat in the previous year, with 4.4% reporting three or more episodes.

Parents or guardians did not know or did not report sore throats about seven times more often than the students (Table 2). Parents or guardians recalled a lower number than the student in 494 cases (14%) and a higher number in 324 cases (9%). In addition, when individual parent/guardian-student pairs were analyzed, only 57% showed perfect agreement between reports (1967 out of 3462 pairs). Cohen’s kappa measure of agreement was estimated to be 0.23 (95% C.I. 0.21–0.25), reflecting a fair agreement between parent or guardian and child for the reported number of sore throats in the past year (Table 3). Younger children were significantly less likely to agree with their parents/guardians on the recall of the number of sore throat episodes; perfect agreement was seen for 53% of pairs when children were ≤12 years old and for 65% of pairs involving students >12 years of age (*p* < 0.0001).

**Table 2:** Reported number of sore throats in year prior to survey

	Reported by student	Reported by parent/guardian
	Frequency (%)	Frequency (%)
Don’t know/missing response	124 (3.58)	890 (25.71)
Zero	2503 (72.30)	1914 (55.29)
One or two	675 (19.50)	505 (14.59)
Three or more	160 (4.62)	153 (4.42)
Total	3462 (100)	3462 (100)

**Table 3:** Agreement between number of pharyngitis episodes reported by student and parent/guardian

	Parent/Guardian				
Student	Don’t know/missing response	0	1–2	3	Total
Don’t know/missing response	**58**	43	16	7	124
0	634	**1611**	205	53	2503
1–2	160	224	**248**	43	675
3	38	36	36	**50**	160
**Total**	890	1914	505	153	3462

Boldfaced numbers indicate where there was perfect agreement between the parent/guardian and student reports regarding number of sore throats in the previous year

### Predictors of reported pharyngitis episodes

A regression analysis with stepwise variable selection was performed to identify predictors of the number of reported sore throats among the following variables: age, gender, weight, and height. The only variable that strongly correlated with reports by both students and parents or guardians was gender. Female students reported on average 0.13 more yearly episodes than male students (0.38 vs. 0.25, *p* < 0.0001). Similarly, parents or guardians reported 0.08 more yearly episodes for their daughters versus for their sons (0.35 vs. 0.27, *p* = 0.0036).

### Reported treatment methods and locations

Treatment practices for pharyngitis were collected based on reports by the parent or guardian. Of the 658 parents or guardians who reported at least one episode of sore throat in the past year for their child, 609 also indicated treatment practices. Of those, 180 students (30% of cases where reports were available) received no treatment for their pharyngitis episode(s), while 429 students (70% of cases where reports were available) received treatment. Treatment on three or more separate occasions was reported for 93 students (15% of cases where reports were available). The majority of reported treatments were received in government clinics, and the second most common site of treatment was at home, presumably with holistic therapies (Table 4). According to reports by the parent or guardian, 216 (50%) of the 429 students who were treated for sore throat in the previous year received an antibiotic. There was no significant difference in the proportion of sick boys or girls who received treatment (67% vs 72%, respectively; *p* = 0.1975). Consistent with the finding that more pharyngitis episodes were recalled for girls, however, was that about two-thirds of the total individuals who had been treated were girls (284 girls; 66%).

**Table 4:** Reported locations of treatment for pharyngitis

Treatment location	N (%)
Government clinic	157 (36.6%)
Home	113 (26.3%)
Private clinic	66 (15.4%)
Hospital	57 (13.3%)
Chemist/pharmacist	47 (11.0%)
Traditional healer	1 (0.2%)
No location specified/Other	25 (6%)
Total number of treatments	466 (100%)

Note that the total number of treatments (and number of treatment locations) is higher than the total number of students treated

### Comparison of reported and measured number of pharyngitis episodes

At time of screening, 77 students (2.2%) reported sore throat, while 19 students (0.5%) had signs visible on physical examination (e.g., pharyngeal erythema, swelling, or exudate) that were severe enough for the student to be referred for follow-up. Based on the average yearly number of sore throats reported by students (0.32) and average symptom duration of 1 week per episode, the expected occurence would be 0.6% of students with sore throat on a given day. This prediction closely matched the measured number of clinically-apparent sore throats, but underestimated the total reported sore throats by about four-fold.

When considering all sore throats reported at screening, there was no difference in mean age between children reporting symptoms and the mean age of all participants (both 14.1 years of age). More than double the number of girls reported sore throats (52) compared to boys (25).

## Discussion

To our knowledge this is the largest epidemiological survey investigating the frequency of pharyngitis in an RHD endemic region in Africa as reported by school-aged children or adolescents and their families, and the first such study in Zambia. Understanding public perceptions and behaviors related to sore throats has substantial implications for the prevention of ARF and RHD. Table 5 lists a summary of key findings and the associated practical implications for future public awareness and education activities in Zambia.

**Table 5:** Summary of key findings and implications for public awareness and education activities to reduce rheumatic heart disease in Zambia

Findings	Implications
Sore throat is perceived to be common in children and adolescents in Zambia	Public awareness messaging does not need to establish that sore throat is a problem locally; rather, the focus can be on validating public perception and educating on best practices for clinical management
Parents/guardians and children have differing perceptions of the frequency of sore throat in children in Zambia	Improved parent/guardian awareness may be needed to ensure vigilance in surveillance for sore throat in children
Sore throat is reported more frequently in girls in Zambia compared with boys	Investigators should explore potential cultural influences during educational activities involving families and providers
Sore throat in children in Zambia is commonly not assessed by a skilled practitioner, which risks sub-optimal treatment	Educating the public on need to seek skilled care for assessment of pharyngitis is a main priority
Some children in Zambia have signs of potential bacterial pharyngitis while attending school	There may be potential opportunity to improve capability at schools to detect and manage sore throat

Pharyngitis was found to be common in Zambian students. Approximately one in five students reported suffering from a sore throat in the previous year and nearly 5% reported having three or more episodes. According to student reports, this equated to an average of 0.32 sore throats per student per year. In Zambia the relative frequency of GAS pharyngitis among all episodes of sore throats (versus pharyngitis due to other etiologies such as viruses) is unknown, but recent studies involving children from other parts of sub-Saharan Africa reveal GAS isolates in 25.5% (in Mali) and 21.6% (in South Africa) of sore throat episodes [22, 23]. Applying the same approximate percentage to the survey data obtained in Zambia gives a GAS pharyngitis attack rate of 0.08 cases per student-year, which is consistent with other studies globally albeit lower than has been previously found or predicted in developing countries [24, 25]. The population in Zambia is 17 million and about a third of the population is aged 5–15 [26]. Using the data obtained in this study, it would therefore be expected that an estimated 440,000 school-aged children in Zambia are affected by GAS pharyngitis annually. This compares reasonably with global estimates of 600 million cases each year [24].

Survey data in this study revealed that parents/guardians and children often did not agree on the number of pharyngitis episodes in the past year. More than a quarter of parents or guardians (26%) did not know or did not report how many pharyngitis episodes their child had experienced, and 14% of parents or guardians reported a lower number of pharyngitis episodes compared with their child. This finding has possible implications for demand-side barriers to proper treatment of GAS pharyngitis since school-aged children would naturally depend on a caregiver to help them seek medical attention for a sore throat. Future work will aim to better understand factors that underlie the discordance.

The number of sore throats was reported in this investigation to be approximately 50% higher in females compared with males, a finding that was consistent in both survey groups (students and parents or guardians). Furthermore, two-thirds of the total number of students treated for pharyngitis were girls. There have been anecdotal reports that GAS pharyngitis is more common in females [27, 28], and several datasets demonstrate higher rates of ARF or RHD in females compared with males [8, 29–32]. It is not understood if this pattern is due simply to sampling biases, differential access to health services by women and girls [31], increased risk of exposure to GAS (e.g., increased participation in childcare activities), increased biological risk for acquisition of GAS-related diseases, or other reasons [1]. The findings of this study suggest the possibility that sore throat frequency could be an influencing factor in the higher prevalence of RHD in females, and underscore the importance of educating girls and their families about treatment of bacterial pharyngitis.

Crucial to the prevention of RHD is appropriate diagnosis and treatment of bacterial pharyngitis with an antibiotic. It was an encouraging finding of this study that families of students who sought treatment for pharyngitis often did so at a site where skilled management of sore throat could presumably take place—that is, at a government clinic, private clinic, or hospital. Still, many episodes of pharyngitis were reported to receive no treatment or to have been treated at the student’s home. In the local context, this generally meant non-prescription treatment, such as gargling with salt water. Less than one-third of students who had pharyngitis, and half of students who had been treated for pharyngitis, received an antibiotic. This rate of antibiotic use may be appropriate overall given that GAS pharyngitis is estimated to account for 20–30% of cases of sore throat in children [22, 23, 33]. However, the degree to which antibiotics are administered to those students most likely to have GAS pharyngitis (compared with viral pharyngitis) is unknown. In Zambia, resource limitations preclude routine bacterial culture or antigen-based testing. Additional operational research is therefore needed to assess health workers’ ability to differentiate likely bacterial from viral pharyngitis using clinical criteria, as well as to better understand their knowledge and skills in treating suspected bacterial sore throat.

A limitation of this study was its reliance on reporting by students and their caregivers (parents or guardians). This investigation was designed to reveal current public perceptions and practices related to sore throat and as such did not incorporate methodologies for bacterial surveillance investigation using cultures or rapid diagnostic tests. Therefore it was not possible to distinguish between viral and bacterial etiologies of pharyngitis. Another limitation is that participants were asked to recall clinical manifestations that occurred over the past year, which could be challenging for some respondents, including young children and caregivers that did not live full-time with their children. This could have contributed to the discordance between the reports of students and parents or guardians. Indeed, agreement in the number of pharyngitis episodes reported by students and parents/guardians was lower for children up to 12 years of age. While the study was not designed to provide a precise measure of sore throat frequency, the data gave some indication of the incidence of sore throat in the population and more importantly describe public perceptions that are essential to the design of public health interventions [19]. Notably, the total prevalence of sore throat at the time of screening was found to be much higher than predicted based on the survey data obtained. This could indicate that students were more likely to report a minor sore throat (e.g., scratching caused by dust) when asked at the time of screening, and recalled more severe sore throat events when surveyed about the previous year. Another possibility is that students may have recalled minor sore throats from a shorter time frame. In either case, survey data may have underestimated the total burden of pharyngitis episodes, and more closely reflected the incidence of clinically-apparent pharyngitis. Another limitation is that the population studied (students that attend school in Zambia’s capital city) may not be representative of the country as a whole; the incidence of GAS pharyngitis may be higher in other parts of Zambia where access to healthcare is poorer (e.g., rural areas and/or those with relatively lower socioeconomic status). The implication is that the frequency of GAS pharyngitis among school-aged children in Zambia each year may be even greater than the already large estimate presented here.

## Conclusions

Pharyngitis is common among school-aged children and adolescents in Zambia. Combining the results of this study with bacteriologic data from other counties in sub-Saharan Africa suggests that the attack-rate of GAS pharyngitis is conservatively 0.08 per student-year, which translates to more than 400,000 cases annually. Other findings of this study are that parents or guardians appear to have variable knowledge about the frequency of sore throat in their children and tend to report lower frequencies, girls are reported to be affected by pharyngitis more than boys, and management of pharyngitis may be suboptimal for many children (both in terms of percentage of families seeking medical care and type of treatment administered). These results provide important insight into current perceptions and practices related to sore throat in Zambia and will be used in the design of public awareness activities aimed at reducing the risk of RHD.

## Abbreviations

ARF: Acute rheumatic fever
GAS: Group A β-haemolytic *Streptococcus*
RHD: Rheumatic heart disease
